# Supplementary material for: A deep learning approach to multi-fiber parameter estimation and uncertainty quantification in diffusion MRI
Source: Med Image Anal. Author manuscript; Available in PMC 2026 May 19. (PMC13186180; doi:10.1016/j.media.2025.103537)
Supplement: 1 [file NIHMS2172423-supplement-1.pdf]

SUPPLEMENTAL MATERIAL

S1. Proof of Theorem 1

Proof. Let $\boldsymbol{P}_V := (\boldsymbol{p}_1, ..., \boldsymbol{p}_V) \subset \mathbb{S}^2$ be a high resolution spherical grid. Since the signal estimates are formed independently between b-values, without loss of generality, we assume $L = 1$ and drop the subscript L notation for clarity. Given rotation matrix $\boldsymbol{R} \in \mathbb{SO}(3)$, we define the rotations

$$\boldsymbol{g} = (g(\boldsymbol{p}_1), ..., g(\boldsymbol{p}_V)), \qquad T_{\boldsymbol{R}}[\boldsymbol{g}] = (g(\boldsymbol{R}^{-1}\boldsymbol{p}_1), ..., g(\boldsymbol{R}^{-1}\boldsymbol{p}_V))$$
$$\widehat{\boldsymbol{f}} = (\widehat{f}(\boldsymbol{p}_1), ..., \widehat{f}(\boldsymbol{p}_V)), \qquad T_{\boldsymbol{R}}\left[\widehat{\boldsymbol{f}}\right] = (\widehat{f}(\boldsymbol{R}^{-1}\boldsymbol{p}_1), ..., \widehat{f}(\boldsymbol{R}^{-1}\boldsymbol{p}_V))$$
$$\boldsymbol{\mathcal{A}}(\widehat{\boldsymbol{f}}) = (\mathcal{A}(\widehat{\boldsymbol{f}})(\boldsymbol{p}_1), ..., \mathcal{A}(\widehat{\boldsymbol{f}})(\boldsymbol{p}_V)), \qquad T_{\boldsymbol{R}}\left[\boldsymbol{\mathcal{A}}(\widehat{\boldsymbol{f}})\right] = (\mathcal{A}(\widehat{\boldsymbol{f}})(\boldsymbol{R}^{-1}\boldsymbol{p}_1), ..., \mathcal{A}(\widehat{\boldsymbol{f}})(\boldsymbol{R}^{-1}\boldsymbol{p}_V)),$$

and $T_{\boldsymbol{R}}^{-1}$ is the inverse transformation defined similarly. To show the desired rotational equivariance, we must show that

$$T_{\boldsymbol{R}}\widehat{\boldsymbol{\mathcal{A}}}(\widehat{\boldsymbol{f}}) = \widehat{\boldsymbol{\mathcal{A}}}(T_{\boldsymbol{R}}\left[\widehat{\boldsymbol{f}}\right]).$$

holds.

First, we note that the prior of $p(\boldsymbol{g})$ is rotationally invariant, i.e. $p(\boldsymbol{g}) = p(T_{\boldsymbol{R}}[\boldsymbol{g}])$. To see this notice that, for any n, the joint prior distribution of the $\boldsymbol{m}^{(n)}$ outlined in Section 2.4 only depends on the angle between them. Clearly, these angles are preserved under rotation, and hence the rotational invariance of the prior follows.

Assuming no regularization, it is easy to see that the distribution of the harmonic coefficient estimator is given by:

$$\widehat{\boldsymbol{c}} \sim \mathcal{N}(\boldsymbol{c}, \sigma_e^2 [\boldsymbol{\Phi}_V^{\intercal}\boldsymbol{\Phi}_V]^{-1}),$$

where $\boldsymbol{c}$ is the true harmonic coefficient vector and $\boldsymbol{\Phi}_V \in \mathbb{R}^{V\times K}$ is the spherical harmonic basis evaluation matrix over the grid $\boldsymbol{P}_V$. For simplicity, we assume K is taken large enough to assume a negligible truncation bias (i.e., signal is approximately band-limited). We assume that V is large enough so that $\boldsymbol{\Phi}_V^{\intercal}\boldsymbol{\Phi}_V \approx V\boldsymbol{I}_K$, which follows due to the orthogonality of the harmonics, since for large V the (k, k') element of $V^{-1}\boldsymbol{\Phi}_V^{\intercal}\boldsymbol{\Phi}_V$ is

$$\frac{1}{V}\sum_{v=1}^{V}\phi_k(\boldsymbol{p}_v)\phi_{k'}(\boldsymbol{p}_v) \approx \int_{\mathbb{S}^2}\phi_k(\boldsymbol{p})\phi_{k'}(\boldsymbol{p})d\boldsymbol{p} = \mathbb{I}\{k = k'\}.$$

These assumption imply the following (approximate) function space distribution of the signal estimator:

$$\widehat{\boldsymbol{f}} \sim \mathcal{N}\left((f(\boldsymbol{p}_1), ..., f(\boldsymbol{p}_V))^{\intercal}, \frac{\sigma_e^2}{V}\boldsymbol{\Phi}_V\boldsymbol{\Phi}_V^{\intercal}\right). \tag{S.1}$$

Let $\tilde{\boldsymbol{u}} = \boldsymbol{R}^{-1}\boldsymbol{u}$ and observe the following identity for the conditional mean:

$$\begin{aligned}
&\mathbb{E}\left[\widehat{f}(\boldsymbol{p})|\boldsymbol{\xi}^{(n)} = \boldsymbol{\xi}^{(n)}, n = n, g = T_{\boldsymbol{R}}[g]\right] = \int_{\mathbb{S}^2} \sum_{i=1}^{n} h_{\mathcal{G}}(\boldsymbol{p}^{\intercal}\boldsymbol{u}|b, \boldsymbol{\xi}_i) g(\boldsymbol{R}^{-1}\boldsymbol{u}|\boldsymbol{m}_i) d\boldsymbol{u} \\
&= \int_{\mathbb{S}^2} \sum_{i=1}^{n} h_{\mathcal{G}}(\boldsymbol{p}^{\intercal}\boldsymbol{R}\tilde{\boldsymbol{u}}|b, \boldsymbol{\xi}_i) g(\tilde{\boldsymbol{u}}|\boldsymbol{m}_i) d\tilde{\boldsymbol{u}} \\
&= \int_{\mathbb{S}^2} \sum_{i=1}^{n} h_{\mathcal{G}}((\boldsymbol{R}^{-1}\boldsymbol{p})^{\intercal}\tilde{\boldsymbol{u}}|b, \boldsymbol{\xi}_i) g(\tilde{\boldsymbol{u}}|\boldsymbol{m}_i) d\tilde{\boldsymbol{u}} = f(\boldsymbol{R}^{-1}\boldsymbol{p}) \\
&= \mathbb{E}\left[\widehat{f}(\boldsymbol{R}^{-1}\boldsymbol{p})|\boldsymbol{\xi}^{(n)} = \boldsymbol{\xi}^{(n)}, n = n, g = g\right].
\end{aligned} \tag{S.2}$$

Now, consider the covariance matrix of (S.1), whose elements are of the form $[\boldsymbol{\Phi}_V\boldsymbol{\Phi}_V^{\intercal}]_{vv'} = \sum_{k=1}^{K}\phi_k(\boldsymbol{p}_v)\phi_k(\boldsymbol{p}_{v'})$. We must investigate the properties of this sum. Using traditional notation, denote the set of real spherical harmonics of degree l as: $\mathcal{H}_l := \{Y_{-l}^{l}, ..., Y_l^l\}$ (see Descoteaux et al. (2007) equation 3 for precise definition). The real symmetric basis $\{\phi_1, ..., \phi_K\}$ is formed from a relabeling of $\bigcup_{l=0}^{R}\mathcal{H}_{2l}$, i.e., only even degrees are taken. Then, by the spherical harmonic addition theorem, we have that

$$\sum_{k=1}^{K}\phi_k(\boldsymbol{p}_v)\phi_k(\boldsymbol{p}_{v'}) = \sum_{l=0}^{R}\sum_{m=-21}^{2l} Y_m^{2l}(\boldsymbol{p}_v)Y_m^{2l}(\boldsymbol{p}_{v'}) \propto P_{2l}(\langle \boldsymbol{p}_v, \boldsymbol{p}_{v'}\rangle),$$

where P_l is the Legendre polynomial of degee l. Crucially, this sum is rotationally invariant, which is inherited from the invariance of the inner product: $\langle \boldsymbol{p}_v, \boldsymbol{p}_{v'}\rangle = \langle \boldsymbol{R}^{-1}\boldsymbol{p}_v, \boldsymbol{R}^{-1}\boldsymbol{p}_{v'}\rangle$. Hence, $\boldsymbol{\Phi}_V\boldsymbol{\Phi}_V^{\intercal}$ is invariant with respect to any rotation of the grid $\boldsymbol{P}_V$. Coupling this property with (S.2), it follows that the vectorized distribution $\boldsymbol{f}$ is rotationally equivariant in the sense

$$p(\widehat{\boldsymbol{f}}|\boldsymbol{\xi}^{(n)}, n, T_{\boldsymbol{R}}[\boldsymbol{g}]) = p(T_{\boldsymbol{R}}\left[\widehat{\boldsymbol{f}}\right]|\boldsymbol{\xi}^{(n)}, n, \boldsymbol{g}).$$

Integrating both sides w.r.t. $p(\boldsymbol{\xi}^{(n)}, n)$, this in turn implies the marginal condition

$$p(\widehat{\boldsymbol{f}}|T_{\boldsymbol{R}}[\boldsymbol{g}]) = p(T_{\boldsymbol{R}}\left[\widehat{\boldsymbol{f}}\right]|\boldsymbol{g}).$$

Now, given that

$$\begin{aligned}
\frac{1}{V}\|T_{\boldsymbol{R}}[\boldsymbol{g}] - T_{\boldsymbol{R}}\left[\boldsymbol{\mathcal{A}}(\widehat{\boldsymbol{f}})\right]\|_2^2 &\approx \int_{\mathbb{S}^2}(g(\boldsymbol{R}^{-1}\boldsymbol{p}) - \mathcal{A}(\widehat{\boldsymbol{f}})(\boldsymbol{R}^{-1}\boldsymbol{p}))^2 d\boldsymbol{p} \\
&= \int_{\mathbb{S}^2}(g(\boldsymbol{p}) - \mathcal{A}(\widehat{\boldsymbol{f}})(\boldsymbol{p}))^2 d\boldsymbol{p} \\
&\approx \frac{1}{V}\|\boldsymbol{g} - \boldsymbol{\mathcal{A}}(\widehat{\boldsymbol{f}})\|_2^2,
\end{aligned}$$

we have that the $\|\cdot\|_2$ norm is approximately invariant to $T_{\boldsymbol{R}}$.

Putting this all together, we have

$$\begin{aligned}
&\int\int \|\boldsymbol{g} - \boldsymbol{\mathcal{A}}(\widehat{\boldsymbol{f}})\|_2^2 p(\widehat{\boldsymbol{f}}|\boldsymbol{g})p(\boldsymbol{g})d\widehat{\boldsymbol{f}}d\boldsymbol{g} \\
&\overset{\text{prior invariance}}{=} \int\int \|T_{\boldsymbol{R}}[\boldsymbol{g}] - \boldsymbol{\mathcal{A}}(\widehat{\boldsymbol{f}})\|_2^2 p(\widehat{\boldsymbol{f}}|T_{\boldsymbol{R}}[\boldsymbol{g}])p(\boldsymbol{g})d\widehat{\boldsymbol{f}}d\boldsymbol{g} \\
&\overset{\text{model equivariance}}{=} \int\int \|T_{\boldsymbol{R}}[\boldsymbol{g}] - \boldsymbol{\mathcal{A}}(T_{\boldsymbol{R}}\left[\widehat{\boldsymbol{f}}\right])\|_2^2 p(T_{\boldsymbol{R}}\left[\widehat{\boldsymbol{f}}\right]|\boldsymbol{g})p(\boldsymbol{g})d\widehat{\boldsymbol{f}}d\boldsymbol{g} \\
&\overset{\text{norm invariance}}{=} \int\int \|\boldsymbol{g} - T_{\boldsymbol{R}}^{-1}\boldsymbol{\mathcal{A}}(T_{\boldsymbol{R}}\left[\widehat{\boldsymbol{f}}\right])\|_2^2 p(T_{\boldsymbol{R}}\left[\widehat{\boldsymbol{f}}\right]|\boldsymbol{g})p(\boldsymbol{g})d\widehat{\boldsymbol{f}}d\boldsymbol{g}
\end{aligned} \tag{S.3}$$

Hence, if $\widehat{\boldsymbol{\mathcal{A}}}$ minimizes (10), it must also minimize

$$\int\int \|\boldsymbol{g} - T_{\boldsymbol{R}}^{-1}\boldsymbol{\mathcal{A}}(T_{\boldsymbol{R}}\left[\widehat{\boldsymbol{f}}\right])\|_2^2 p(T_{\boldsymbol{R}}\left[\widehat{\boldsymbol{f}}\right]|\boldsymbol{g})p(\boldsymbol{g})d\widehat{\boldsymbol{f}}d\boldsymbol{g}.$$

which in turn implies the risk minimizer must also satisfy

$$\widehat{\boldsymbol{\mathcal{A}}}(\widehat{\boldsymbol{f}}) = T_{\boldsymbol{R}}^{-1}\widehat{\boldsymbol{\mathcal{A}}}(T_{\boldsymbol{R}}\left[\widehat{\boldsymbol{f}}\right]),$$

which gives the equivariance condition

$$T_{\boldsymbol{R}}\widehat{\boldsymbol{\mathcal{A}}}(\widehat{\boldsymbol{f}}) = \widehat{\boldsymbol{\mathcal{A}}}(T_{\boldsymbol{R}}\left[\widehat{\boldsymbol{f}}\right])$$

as desired. □

S2. Simulation Algorithms

Algorithm S2 provides pseudo-code for sampling from the joint distribution of diffusion signals and model parameters. Algorithm S3 provides pseudo-code for sampling from the joint distribution of diffusion signal function estimates and ODF. Algorithm S4 provides pseudo-code for sampling from the joint distribution of biophysical kernel parameters and estimated demixxed signal curves.

S3. Additional Methodological Details

S3.1. Signal Demixxing

S3.1.1. Monotonic Spline Parameterization

We note that the functions $\bar{h}_{\mathcal{G}}^{(i,l)}$'s are monotonic decreasing in t_i^*. In order to enforce this property in our estimation, we parameterize them using basis expansion over monotonic decreasing cubic b-splines of rank J, constructed by following the formulation in Pya and Wood (2015). For completeness, we provide salient details on the construction of this spline model, and refer the interested reader to the reference for a more thorough treatment. Define $\boldsymbol{\gamma}(t) = (\gamma_1(t), ..., \gamma_J(t))$ to be the rank J cubic b-spline basis. We model $\bar{h}_{\mathcal{G}}^{(i,l)}(t_i^*) \approx$

Algorithm S2 Sample from $p(\boldsymbol{S}, \boldsymbol{\xi}^{(n)}, \boldsymbol{m}^{(n)})$

1: **Input**: Sampling design $\boldsymbol{P}_{M,l}$, b_l for $l = 1, ..., L$, measurement error variance σ_e^2
2: Sample $n \sim \text{Unif}([1, ..., n_{max}])$
3: Sample $(z_{1,1}, z_{1,2}, z_{2,1}, ..., z_{n,1}, z_{n,2})$ using rejection sampling with a proposal Dirichlet$((\frac{1}{2n}, ..., \frac{1}{2n}))$ and rejection criteria $z_{i,1} < 0.1$ for any $i = 1, ..., n$.
4: For $i = 1, ..., n$, independently sample $(D_{a,i}, D_{e,i}^{||}, D_{e,i}^{\perp}) \sim \text{Unif}(\Xi)$ using rejection sampling with a box-uniform proposal, rejecting any samples that don't respect the polytope constraints.
5: Form $\boldsymbol{\xi}_i = (D_{a,i}, D_{e,i}^{||}, D_{e,i}^{\perp}, z_{i,1}, z_{i,2})$ for $i = 1, ..., n$
6: Sample $(\boldsymbol{m}_1, ..., \boldsymbol{m}_n)$ using rejection sampling with a proposal $\prod_{i=1}^{n} \text{Unif}(\mathbb{S}_+^2)$ and rejection criteria defined by the crossing angle constraints outlined in Section 2.4
7: Simulate $\boldsymbol{S} = (\boldsymbol{s}_1, ..., \boldsymbol{s}_L)$ from (7)
8: **return** $(\boldsymbol{S}, \boldsymbol{\xi}^{(n)}, \boldsymbol{m}^{(n)})$

Algorithm S3 Sample from $p(\widehat{\boldsymbol{f}}_L, \boldsymbol{g})$

1: **Input**: High-resolution spherical grid points $\boldsymbol{P}_V = \{\boldsymbol{p}_1, ..., \boldsymbol{p}_V\}$.
2: Sample $(\boldsymbol{S}, \boldsymbol{\xi}^{(n)}, \boldsymbol{m}^{(n)})$ via Algorithm S2
3: Compute $\widehat{f}_L$ via (9) using simulated $\boldsymbol{S}$
4: Set $\boldsymbol{g} = \frac{1}{n} \sum_{i=1}^{n} g_i(\boldsymbol{p}_v | \boldsymbol{m}_i)$, $v = 1, ..., V$
5: Set $\widehat{\boldsymbol{f}}_L$ as the evaluations of $\widehat{f}_L$ over $\boldsymbol{P}_V$
6: **return** $(\widehat{\boldsymbol{f}}_L, \boldsymbol{g})$

Algorithm S4 Sample from $p(\boldsymbol{\xi}_i, \widehat{\overline{\boldsymbol{h}}}_{\mathcal{G}}^{(i)} | n)$

1: Sample $(\boldsymbol{S}, \boldsymbol{\xi}^{(n)}, \boldsymbol{m}^{(n)})$ via Algorithm S2
2: Compute $\boldsymbol{t}_{l,m}^{*} = ((\boldsymbol{p}_{l,m}^{\intercal} \boldsymbol{m}_1)^2, ..., (\boldsymbol{p}_{l,m}^{\intercal} \boldsymbol{m}_n)^2)$ and $\boldsymbol{\Gamma}_{mj}^{(i,l)} = \gamma_j(t_{l,m,i}^{*})$, for $i = 1, ..., n$, $l = 1, ..., L$, $m = 1, ..., M$
3: Estimate $\widehat{\boldsymbol{a}}^{(1,l)}, ..., \widehat{\boldsymbol{a}}^{(n,l)}$ via (16) for $l = 1, ..., L$
4: Form $\widehat{\overline{\boldsymbol{h}}}_{\mathcal{G}}^{(i)}(t_i^{*}) = (\boldsymbol{\gamma}(t_i^{*})^{\intercal} \widehat{\boldsymbol{a}}^{(i,1)}, ..., \boldsymbol{\gamma}(t_i^{*})^{\intercal} \widehat{\boldsymbol{a}}^{(i,L)})$
5: Return $\{\widehat{\overline{\boldsymbol{h}}}_{\mathcal{G}}^{(i)}, \boldsymbol{\xi}_i, n\}$, $i = 1, ..., n$

$\boldsymbol{\gamma}(t_i^{*})^{\intercal} \boldsymbol{a}^{(i,j)}$, where $\boldsymbol{a}^{(i,j)} := \boldsymbol{\Sigma} \tilde{\boldsymbol{\beta}}^{(i,j)}$, $\tilde{\boldsymbol{\beta}}^{(i,l)} = (\beta_1^{(i,l)}, \exp(\beta_2^{(i,l)}), ..., \exp(\beta_J^{(i,l)}))$ with $\boldsymbol{\beta}^{(i,l)} = (\beta_1^{(i,l)}, ..., \beta_J^{(i,l)})$, and $\boldsymbol{\Sigma} \in \mathbb{R}^{J \times J}$ defined element-wise as

$$\boldsymbol{\Sigma}_{jj'} = \begin{cases} 0 \text{ for } j < j' \\ 1 \text{ for } j' = 1, j \geq 1 \\ -1 \text{ for } j' \geq 2, j \geq j'. \end{cases}$$

It can be validated that modeling the basis expansion coefficients in this way guarantees the desired monotonicity property in the estimates. The optimization problem (16) can be

written in the new parameterization as follows:

$$\widehat{\mu}_l, \widehat{\boldsymbol{\beta}}^{(1,l)}, ..., \widehat{\boldsymbol{\beta}}^{(n,l)} = \min_{\mu_l, \boldsymbol{\beta}^{(1,l)}, ..., \boldsymbol{\beta}^{(n,l)}} \left\| \boldsymbol{s}_l - \left[\mathbf{1}_M \mu_l + \sum_{i=1}^{n} \boldsymbol{\Gamma}^{(i)} \boldsymbol{\Sigma} \tilde{\boldsymbol{\beta}}^{(i,l)} \right] \right\|_2^2 \quad \text{s.t.} \quad \mathbf{1}_M^{\intercal} \boldsymbol{\Gamma}^{(i)} \boldsymbol{\Sigma} \tilde{\boldsymbol{\beta}}^{(i,l)} = 0, \text{ for } i = 1, ..., n, \tag{S.4}$$

and (approximately) solved using the Newton's method solver proposed in Pya and Wood (2015).

S3.1.2. A Brief Note on Statistical Properties

Recall that the signal demixing approach outlined in Section 3.2.1 relies on a transformation $\boldsymbol{p} \mapsto \boldsymbol{t}_n^*$, mapping the domain (at each b-shell) $\mathbb{S}^2 \mapsto [0,1]^n$. This transformation is designed to exploit the *additive structure* of the signal function in the transformed space. As shown in Figure 3 of the main text for $n = 2$, the signal function in this transformed space is a surface over the n-dimensional hyper-cube which is additive in the marginal decay curves. This additive structure provides a way of demixing the signal. Specifically, the kernel parameters of the i'th fiber can be inferred using only the i'th estimated decay curve, under approximate conditional independence.

Optimal estimates of the additive signal curves are known to enjoy good statistical properties, as the statistical errors $\|\widehat{\bar{\boldsymbol{h}}}_{\mathcal{G}}^{(i)} - \bar{\boldsymbol{h}}_{\mathcal{G}}^{(i)}\|$ are (asymptotically in number of samples M) independent of n (Stone, 1985; Horowitz et al., 2006). This implies that the demixxed signal functions can be estimated with a (asymptotically) constant statistical rate regardless of n, which is a result of the special additive structure and is not the case for general n-dimensional functions (which require exponentially more data as n increases). This structure offers a type of protection against the curse of dimensionality for larger n.

Although the convergence of signal demixing-based inference was not explicitly investigated in this work, as M was fixed for all experiments, we suggest this as an interesting direction for future research. Specifically, it would be valuable to evaluate whether the theoretically fast statistical convergence of the decay curve estimates for large n translates into practically faster convergence rates for fiber-specific microstructure compared to, for example, joint optimization in the original space.

S3.2. Computing Marginal HDRs

We form HDRs marginally for each parameter in $\xi_{i,p} \in \boldsymbol{\xi}_i$, as follows. First, we bin the support of $\xi_{i,p}$ into B disjoint intervals. We then draw Q samples from the amortized posterior $\boldsymbol{\xi}_{i,q} \sim p_{\widehat{\eta}}(\boldsymbol{\xi}_i | \widehat{\bar{\boldsymbol{h}}}_{\mathcal{G}}^{(i)}, n)$ and, for each binned sub-interval, we calculate the proportion of samples $\xi_{i,p,q}$ belonging to each bin. The bins are sorted in descending order according to this proportion and the smallest index r in the sorted list such that the cumulative sum of the first r elements is greater than $(1 - \alpha)$ is identified. Finally, the HDR is defined as the union of the bins corresponding to the first r elements of the sorted list. Algorithm S5 provides pseudo-code for this procedure.

Algorithm S5 Forming Highest Density Regions (HDRs) for each parameter

Input: Number of bins B, number of samples Q, the level α
Draw Q samples from the amortized posterior: $\boldsymbol{\xi}_{i,q} \sim p_{\widehat{\eta}}(\boldsymbol{\xi}_i | \widehat{\overline{\boldsymbol{h}}}_{\mathcal{G}}^{(i)}, n)$
for each parameter $\xi_{i,p} \in \boldsymbol{\xi}_i$ **do**
    Bin the support of $\xi_{i,p}$ into B disjoint intervals
    Initialize array *counts* of size B to zero
    for $q = 1, ..., Q$ **do**
        Identify the bin index k for $\xi_{i,p,q}$
        counts$[k]$ = *counts*$[k] + 1$
    end for
    proportions$[k]$ = *counts*$[k]/Q$
    sorted_proportions = sort(*proportions*, order=descending)
    Find the smallest index r such that cumulative sum of *sorted_proportions* up to r is greater than $(1 - \alpha)$
    Define HDR as the union of the bins corresponding to the first r indices in *sorted_proportions*
end for
Output: Return HDR

S4. Additional Experimental Results

S4.1. Synthetic Data

S4.1.1. Additional Details on Estimation for $n \geq 2$

Figures S1 and S2 show density colored scatter plots for estimated versus ground truth parameters for all estimators. Consistent with the aggregate results shown in Table 2 of the main text, for the $n = 2$ fiber case, we observe a general increase in estimation errors across all parameters for all estimators compared to the $n = 1$ results. This is reflected in weaker correlations between predictions and ground truth, with density plots appearing more diffuse and/or somewhat tilted relative to the line of equality. For D_a, all estimators exhibit positive correlation with ground truth. However, MLE 1/2 and LFI-MAP all display some examples of significant under estimation near the upper bound of ground truth (non-trivial density in bottom right), while MLE 1/2 also exhibit substantial overestimation near the lower bound of ground truth (non-trivial density in upper left). This behavior likely arises from increased multi-modality in this higher-dimensional case versus $n = 1$. In contrast, the D_a estimates of LFI-PM do not exhibit such extreme errors at either boundary. Instead, we observe a relatively minor "tendency toward the interior" behavior (as noted for $D_e^{||}$ in the $n = 1$ case in the main text), with modest over- and underestimation at the boundaries compared to the other estimators. We speculate this is due to the averaging effect of the posterior mean computation mitigating extreme errors that result from selecting an inaccurate mode, and could be desirable behavior in this regime. For $D_e^{||}$, LFI-MAP is the only estimator producing somewhat reasonable estimates, though the density plot appears more diffuse relative to the line of equality compared to the $n = 1$ case, reflecting the increased difficulty of the inverse problem. LFI-MAP and

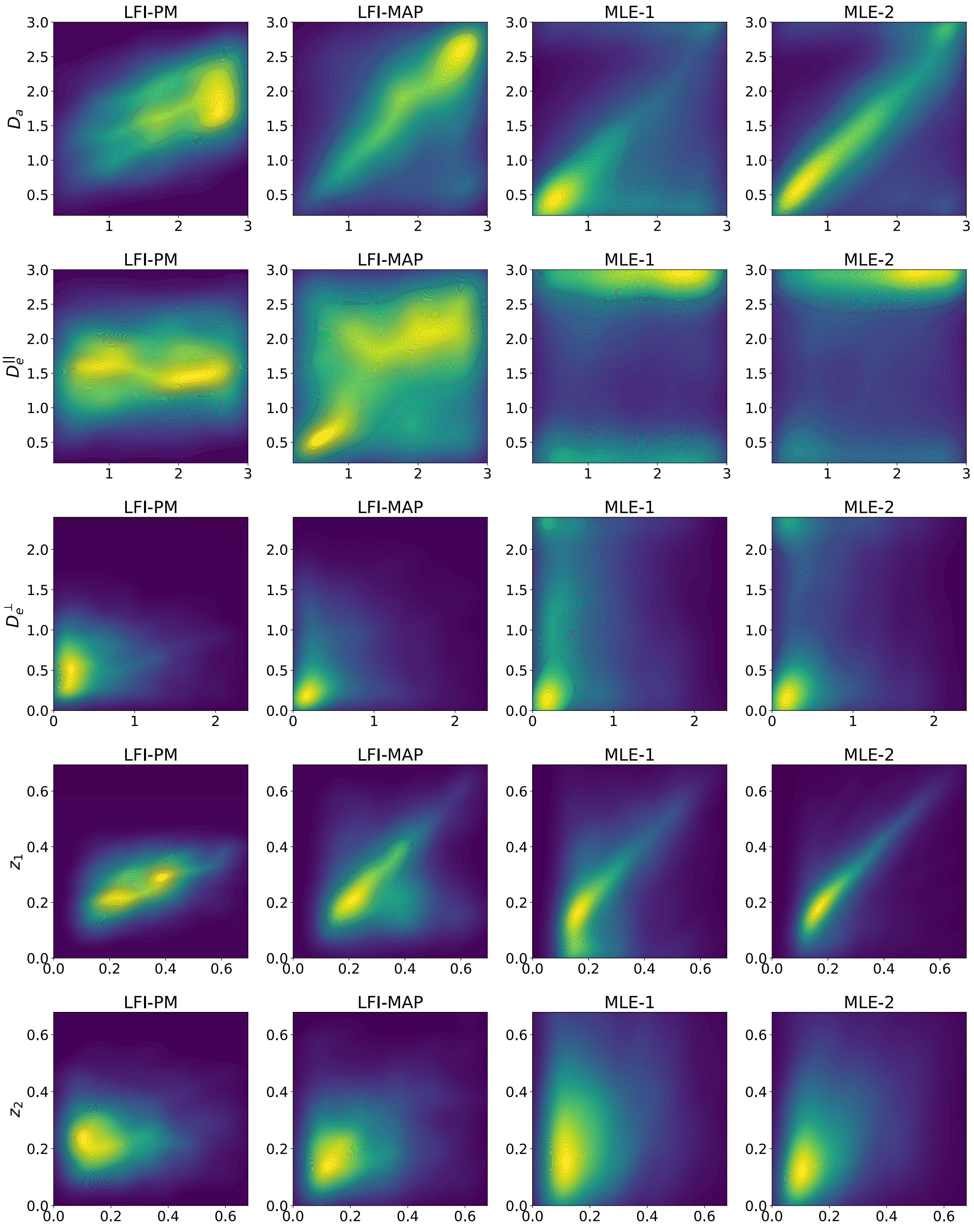

Figure S1: Density-colored scatter plots for the $n = 2$ case showing the relationship between ground truth (x-axis) and estimated parameters (y-axis) for all test-set examples for each method (columns).

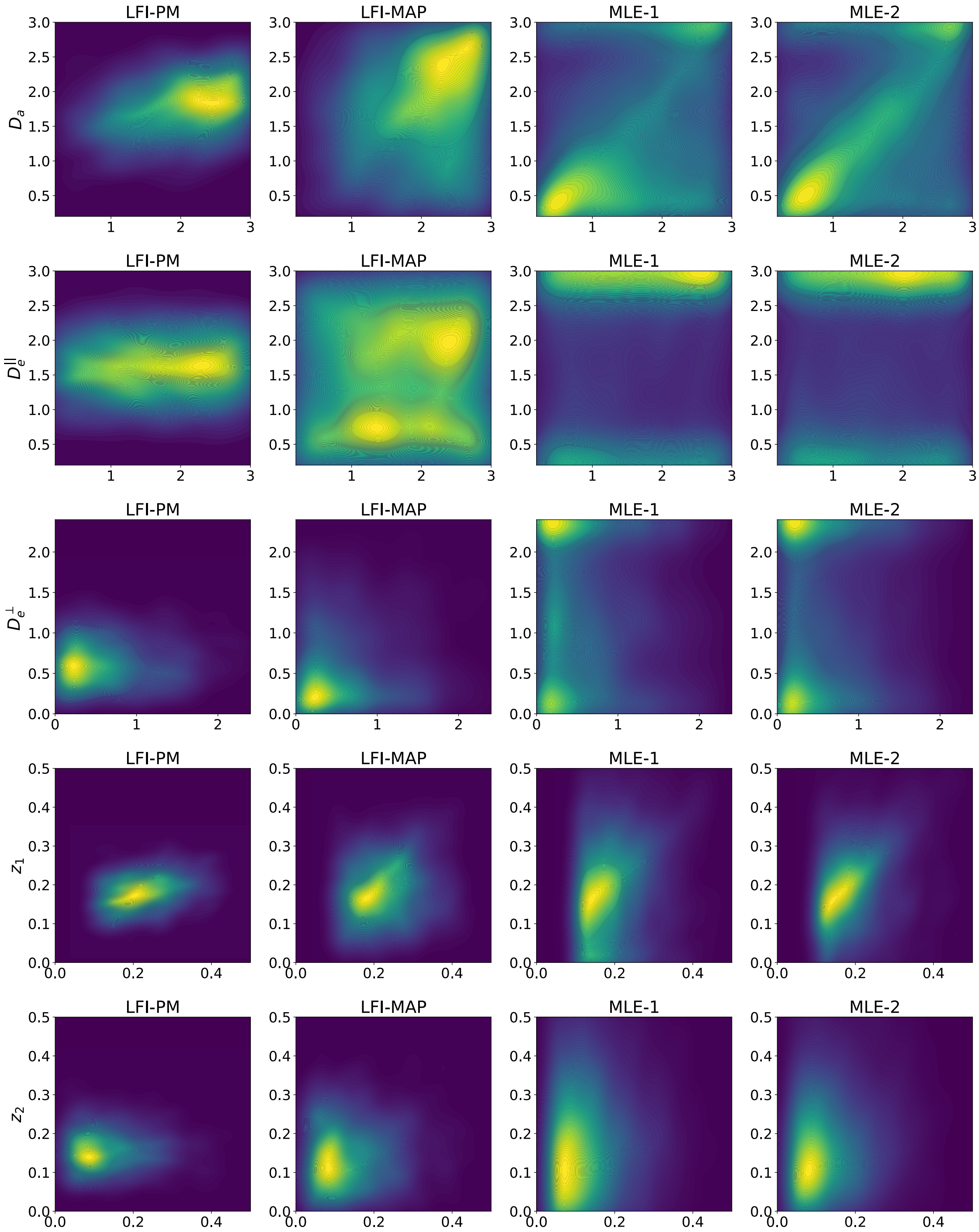

Figure S2: Density-colored scatter plots for the $n = 3$ case showing the relationship between ground truth (x-axis) and estimated parameters (y-axis) for all test-set examples for each method (columns).

LFI-PM perform reasonably well for small ground truth values of $D_e^{\perp}$, but estimation errors increase as the ground truth values increase. The MLE 1/2 estimators both exhibit regions of dramatic overestimation (mode in the top left of Figure S1). All methods do reasonably well for intra-axonal volume fraction, while LFI-MAP appears to have the best performance for z_2, which is backed up by the aggregate results in manuscript Table 2.

For the $n = 3$ fiber case, we observe a further increase in estimation errors across all parameters and estimators compared to the $n = 2$ fiber case, consistent with the aggregate results presented in Table 2 in the main text. The comparison of estimators for D_a is similar to the $n = 2$ case, except for the general trend of increased errors across all estimators. In this regime, estimates of $D_e^{||}$ or $D_e^{\perp}$ from any estimator should be treated with caution, as they are highly unreliable. On the other hand, the methods perform reasonably well for the intra-axonal volume fraction z_1, although MLE 1/2 appear to exhibit greater bias for low values. These findings suggest that, while the signal is very weakly informative for the extra-axonal parameters, it may still be sufficiently informative to allow for usable estimates for the intra-axonal volume fraction and gives some limited information for D_a. This could be beneficial in certain scenarios, such as maintaining accurate tractography in crossing fiber situations, where matching intra-axonal volume fractions could help identify and select the appropriate peak.

S4.1.2. Comparison With Trivial Prior Mean Estimator

The biologically constrained uniform prior over model parameters outlined in Section 2.4 was chosen deliberately as a non-informative prior to avoid biasing inference. In the case when the signal provides little to no information about the parameters of interest, we would expect our approximate Bayesian posterior to essentially return the prior. Hence, we can define the "trivial estimator" as simply taking the prior mean. This estimator is unbiased, but minimally informative in the sense that it is not using any information in the signal to update its estimate (just returning the prior). As such, it serves as a type of baseline representing the simplest possible approach. Table S1 shows the average absolute errors of the prior mean estimator over the test set. Comparing these to the results reported in Table 2 of the main text, we make the following observations: First, our inversion algorithm does not merely return the mean of the training distribution for any of the $n = 1, 2$ or 3-fiber cases. Second, our inversion algorithm shows a better relative improvement over the trivial prior mean estimator for the volume fractions (z_1/z_2) and D_a, than it does for the extra-axonal diffusivities, $D_e^{||}, D_e^{\perp}$. This is reflected in the model uncertainty estimates in Table 3, where the high-density regions of $D_e^{||}$ (and to a lesser extent $D_e^{\perp}$) take up a relatively larger portion of the parameter range than the other parameters (quantified by HDR-S), indicating a more spread out posterior distribution. Finally, the prior mean appears to "outperform" some of the competing methods in terms of average error. However, this advantage arises not from containing more information but rather from the substantial bias present in the other estimators.

S4.1.3. Inference Under Forward Model Misspecification

To examine the performance of our model under varying levels of model misspecification, we apply our pre-trained inverter (trained using the forward model from Section 2.1

		D_a	$D_e^{\|\|}$	$D_e^{\perp}$	z_1	z_2
**1-Fiber**	Abs. Error	0.703	0.700	0.399	0.197	–
**2-Fiber**	Abs. Error	0.710	0.697	0.397	0.113	0.116
**3-Fiber**	Abs. Error	0.706	0.701	0.400	0.073	0.074

Table S1: Average absolute error results for the naive prior mean estimator on the synthetic test set.

assuming κ very large and fixed) to test data generated with variable κ. That is, we model the noiseless test signals as

$$f(\boldsymbol{p}, b) \approx \sum_{i=1}^{n} \int_{\mathbb{S}^2} \left[z_{i,1} \exp\left(-bD_{i,a}(\boldsymbol{p}^\intercal \boldsymbol{u})^2\right) + z_{i,2} \exp\left(-bD_{i,e}^{\perp} - b(D_{i,e}^{||} - D_{i,e}^{\perp})(\boldsymbol{p}^\intercal \boldsymbol{u})^2\right) \right] \times C(\kappa_i)\exp(\kappa_i(\boldsymbol{m}_i^\intercal \boldsymbol{u})^2) d\boldsymbol{u}. \tag{S.5}$$

Table S2 shows the performance of both LFI-PM and LFI-MAP estimators, along with uncertainty calibration as measured by the ECP of the 95% posterior HDR, for the $n = 1$ fiber case with $\kappa = 30, 20, 10$, representing increasing levels of misspecification. For convenience, the high-concentration test data results ($\kappa \to \infty$) are also included, copied from Table 2 of the main manuscript. As expected, we see that the performance degrades with decreasing κ, with reasonable results shown for $\kappa = 30$ (and to a lesser extent $\kappa = 20$), while inference for $\kappa = 10$ is quite problematic in both estimation and uncertainty quantification for D_a and $D_e^{||}$.

Similarly, Table S3 shows the results for misspecified inference for the $n = 2$ case obtained by applying the pre-trained inverter to out of distribution test data from both $(\kappa_1, \kappa_2) \sim \text{Unif}([25, 35]^2)$ and $(\kappa_1, \kappa_2) \sim \text{Unif}([10, 15]^2)$. The $\kappa_1, \kappa_2 \to \infty$ (in-distribution) high-concentration 2-fiber test data results from main text Table 2 are also provided for comparison. We observe that the uncertainty remains quite well calibrated for $(\kappa_1, \kappa_2) \sim \text{Unif}([25, 35]^2)$, with all of the ECP relatively close to the nominal 0.95 level. For $(\kappa_1, \kappa_2) \sim \text{Unif}([10, 15]^2)$, the uncertainty is generally underestimated for all parameters, with some reporting only mild over-confidence ($D_e^{\perp}$, z_1), and others significantly under-covered (z_1). As was observed in the $n = 1$ results, the degradation in estimation performance, measured by absolute errors and bias, is significantly more pronounced in the lower κ case. This outcome is expected, as the lower κ represents a greater deviation from the forward model ($\kappa \to \infty$) used to train the posterior estimator. The parameter estimate which suffers the most significant degradation (for both posterior mean and MAP estimators) is intra-axonal volume fraction D_a, both in terms of absolute errors and bias. This could be because both parameters have similar effects on the shape of the signals, i.e., both higher D_a and κ will lead to more anisotropy.

One approach for dealing with (known) model misspecification is to incorporate the additional complexity into the forward model and perform inference on this expanded parameter space. The main contribution of our work is the two-stage approach to parameter inference, specifically designed to exploit the general structure of forward models like those in Equation (2), rather than being restricted to the particular form chosen in Section 2. Therefore, we can easily accommodate inclusion of κ into our framework by replacing

	Metric	D_a	$D_e^{\|\|}$	$D_e^{\perp}$	z
		$\kappa \to \infty$			
LFI-PM	Abs. Error	0.199	0.549	0.275	0.079
	Bias	-0.001	0.021	0.005	0.006
LFI-MAP	Abs. Error	0.133	0.180	0.077	0.039
	Bias	-0.013	0.007	-0.013	0.001
**ECP**		0.953	0.941	0.949	0.953
		$\kappa = 30$			
LFI-PM	Abs. Error	0.296	0.588	0.279	0.138
	Bias	-0.241	0.069	-0.023	-0.082
LFI-MAP	Abs. Error	0.299	0.317	0.117	0.07
	Bias	-0.352	0.408	0.083	-0.026
**ECP**		0.935	0.889	0.901	0.854
		$\kappa = 20$			
LFI-PM	Abs. Error	0.374	0.570	0.270	0.155
	Bias	-0.345	0.103	-0.054	-0.107
LFI-MAP	Abs. Error	0.396	0.315	0.108	0.087
	Bias	-0.449	0.374	0.003	-0.062
**ECP**		0.904	0.861	0.866	0.786
		$\kappa = 10$			
LFI-PM	Abs. Error	0.607	0.640	0.279	0.200
	Bias	-0.620	0.178	-0.029	-0.157
LFI-MAP	Abs. Error	0.678	0.409	0.136	0.140
	Bias	-0.714	0.405	-0.012	-0.101
**ECP**		0.715	0.799	0.773	0.622

Table S2: Biophysical parameter inference for different levels of misspecification (κ values) for the $n = 1$ case.

step 7 in Algorithm S2 and instead generating noiseless signals from (S.5) under the chosen acquisition design and then adding Gaussian noise. To demonstrate this flexibility, Table S4 provides some preliminary results for the $n = 1$ case when κ is included in the forward model under the same HCP-like acquisition scheme. The biological prior Unif($[5, 35]$) is used, and the remainder of the inversion procedure is kept the same. Given these results are averaged over the test set sampled from $\kappa \sim$ Unif($[5, 35]$), we see significant improvement over the corresponding misspecified inference results reported in Table S2 in terms of errors, bias and, importantly, uncertainty quantification, as now the ECP is back near 0.95 for all parameters. When comparing the results of the two estimators, we observed that LFI-MAP generally has lower absolute errors but increased bias compared to LFI-PM. Results from (Jelescu et al., 2016) indicate that the forward model with standard model kernel and Watson density with variable κ exhibits a substantial degree of ill-posedness. This manifests in a multi-modal posterior (and likelihood) with at least two plausible solutions: one tending towards $D_a < D_e^{||}$, low z_1, and high κ, and the other with $D_a > D_e^{||}$, high z_1, and low value of κ. Given this context, the lower-error but higher-bias performance of

		D_a	$D_e^{\|\|}$	$D_e^{\perp}$	z_1	z_2
		$(\kappa_1, \kappa_2) \to \infty$				
LFI-PM	Abs. Error	0.291	0.626	0.345	0.052	0.089
	Bias	-0.045	0.035	-0.010	0.018	-0.018
LFI-MAP	Abs. Error	0.283	0.579	0.334	0.047	0.086
	Bias	-0.045	0.017	-0.012	0.002	-0.007
		$(\kappa_1, \kappa_2) \sim$ **Unif**$([25, 35]^2)$				
LFI-PM	Abs. Error	0.372	0.617	0.353	0.061	0.113
	Bias	0.146	-0.021	-0.020	0.041	-0.051
LFI-MAP	Abs. Error	0.401	0.625	0.341	0.058	0.093
	Bias	0.184	-0.096	0.012	0.034	-0.023
**ECP**		0.938	0.913	0.947	0.943	0.961
		$(\kappa_1, \kappa_2) \sim$ **Unif**$([10, 15]^2)$				
LFI-PM	Abs. Error	0.503	0.622	0.356	0.073	0.129
	Bias	0.314	-0.024	-0.023	0.062	-0.073
LFI-MAP	Abs. Error	0.590	0.663	0.335	0.074	0.106
	Bias	0.420	-0.147	0.007	0.050	-0.049
**ECP**		0.886	0.889	0.925	0.838	0.921

Table S3: Biophysical parameter inference under model misspecification for different κ ranges for the $n = 2$ case.

Table S4: Inference results for $\kappa \sim \text{Unif}([5, 35])$ in the model.

		D_a	$D_e^{\|\|}$	$D_e^{\perp}$	z_1
PM	Abs. Errors	0.3383	0.6432	0.3172	0.1370
	Bias	-0.0087	-0.0071	-0.0135	-0.0067
MAP	Abs. Errors	0.3295	0.4029	0.1823	0.1287
	Bias	0.1500	-0.0860	0.0053	0.0164
**ECP**		0.946	0.9304	0.9454	0.9658

LFI-MAP likely arises from its tendency to select the "wrong mode" for some non-trivial number of test cases. In contrast, LFI-PM averages over the modes, leading to higher errors (as it lands between the modes) but lower bias. These estimators have different properties, and it is likely application dependent which one (if either) a user would want to report. Nonetheless, we emphasize the flexibility of our methodology as a general-purpose solver, capable of adapting to different forward models (and acquisition schemes).

S4.2. Real Data

Figure S3 shows a sub-cluster of part of the corpus callosum bundle extracted from the tractography results using *WhiteMatterAnaylsis*.

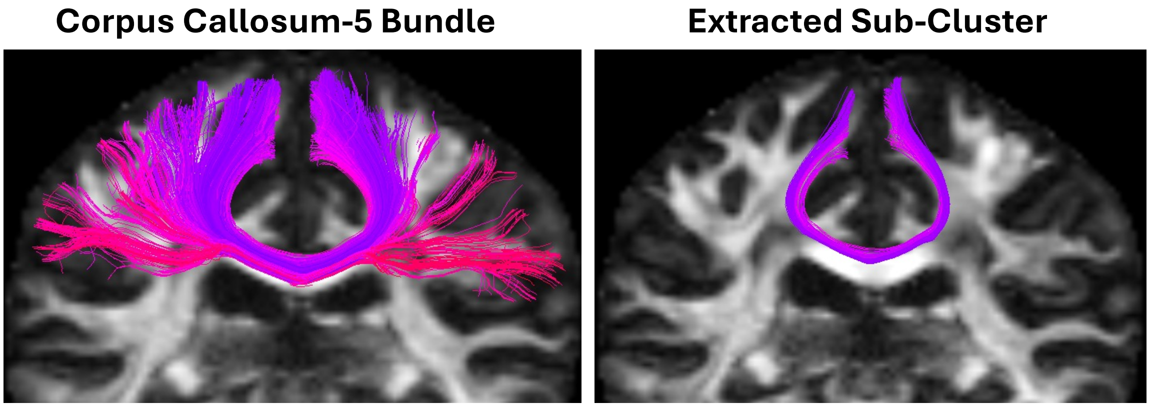

Figure S3: (Left) Corpus Callosum-5 (CC5) anatomical bundle obtained from streamline tractography clustering results using *WhiteMatterAnaylsis* package. (Right) Sub-cluster extracted from CC5 bundle.

REFERENCES

Descoteaux, M., E. Angelino, S. Fitzgibbons, and R. Deriche (2007). Regularized, fast, and robust analytical Q-ball imaging. *Magn Reson Med 58*(3), 497–510.

Horowitz, J., J. Klemelä, and E. Mammen (2006). Optimal estimation in additive regression models. *Bernoulli 12*(2), 271 – 298.

Jelescu, I. O., J. Veraart, E. Fieremans, and D. S. Novikov (2016). Degeneracy in model parameter estimation for multi-compartmental diffusion in neuronal tissue. *NMR in Biomedicine 29*(1), 33–47.

Pya, N. and S. N. Wood (2015). Shape constrained additive models. *Statistics and Computing 25*(3), 543–559.

Stone, C. J. (1985). Additive Regression and Other Nonparametric Models. *The Annals of Statistics 13*(2), 689 – 705.
